# The relation between authoritarian leadership and belief in fake news

**DOI:** 10.1038/s41598-023-39807-x

**Published:** 2023-08-08

**Authors:** Juan Ospina, Gábor Orosz, Steven Spencer

**Affiliations:** 1 Department of Psychology, The Ohio State University, Columbus, USA; 2 ULR 7369-URePSSS-Unité de Recherche Pluridisciplinaire Sport Santé Société, Sherpas, Univ. Lille, Univ. Artois, Univ. Littoral Côte d’Opale, Liévin, France

**Keywords:** Human behaviour, Statistics

## Abstract

Individual factors such as cognitive capacities matter when one is requested to spot fake news. We suggest, however, that social influence—specifically as exercised by an authoritarian leader—might matter more if one is expected to agree with the fake news. We developed a single-item prototype measure of leadership styles and recruited participants from four Western democratic countries (Australia, Canada, United Kingdom, United States, *N* = 501) who identified their immediate boss as an autonomous, paternalistic, or authoritarian leader. Then they were asked to evaluate the accuracy of several fake news articles and their expectations to agree with their boss when asked about these articles. People with authoritarian bosses were less accurate in spotting fake news (Cohen’s *d* = 0.32) compared to employees with autonomous bosses. The bigger effect, however, was that they would agree with their boss about the fake news article when it was shared by their authoritarian boss compared to employees with autonomous (Cohen’s *d* = 1.30) or paternalistic bosses (Cohen’s *d* = 0.70). We argue that in addition to effects on the perceived accuracy of information, social influence, conformity, and obedience are crucial and unacknowledged factors of how misinformation may be maintained and propagated by authoritarian leaders.

## Introduction

Imagine your boss sends you a fake news article on social media. You meet, and they ask you about this article. Would you openly agree with your boss? What would you think of your boss in this situation? We suggest your reactions to this situation would depend on your perceptions of your boss. If you have an autonomous boss who truly listens to you, values your input, and wants you to improve, we suggest you will have confidence and permission to disagree and explain your rationale for why the article is fake. If you have a paternalistic boss who cares about you, and sometimes listens to you, but does not pay that much attention to you, we suggest you have space to respectfully disagree with them (e.g., agree to disagree) or agree with them and avoid conflict, but your job would not be at risk. If you have an authoritarian boss who does not care about your ideas, does not value your input, and only wants your compliance, however, we suggest that you will feel strong social pressure to agree with the fake news and most people will agree even when they know full well that it is fake. After all, if you disagree, you lose your job.

Although past research has primarily focused on the cognitive and motivational mechanisms of why people fail to reject misinformation, we suggest that the social forces in our everyday lives are a large and understudied source of why people agree with fake news. We examine how the leadership style of a superior is a key social factor that explains why people agree with fake news.

Past research has mostly found different mechanisms that people may use to distinguish real from fake news: cognitive processing (analytic thinking^1–3^, digital literacy^4^), motivational^5^, and partisanship^6–12^ explanations. All of these factors matter when trying to understand how people distinguish real from fake news because they explain how people can develop individual skills to identify fake news. We suggest, however, that social factors are at least as important in people’s agreement with fake news. Specifically, people are often less concerned about being accurate in distinguishing real from fake news, and more concerned about agreeing with important people that control their life. In these situations, the cognitive, motivational, and demographic factors are less important. What truly matters, we argue, is whether you agree with those who have power over you when you know they will wield it against you.

We suggest that there are at least three forms of social influence that powerfully create agreement with fake news when wielded by an authoritarian boss: pressures to conform, obedience to authority, and expected real-life negative consequences (e.g., punishment). The higher the magnitude of these three factors, the more people would conform to believing misinformation. In the classic Asch study of conformity^13^, a large number of people conform to judgments they know are wrong when faced with a unanimous majority, demonstrating that pressure to conform can lead people to such agreement. As Stanley Milgram demonstrated, an authoritarian leader can ramp up acquiescence to decisions that people know are wrong and these powerful findings occurred even when people knew the authoritarian leader had no control over any important part of their life^14^. How much more will authoritarian leaders who can punish people in ways that are important to them create an agreement with their errant views? We suggest that the powerful forces of conformity, obedience, and fear of punishment will work together to cause people to agree with authoritarian leaders even when they know the information they are promoting is inaccurate and wrong. That is, people will agree with fake news when an authoritarian leader promotes such information.

Past research has alluded to different leadership styles that are closely aligned with our leadership prototypes. Lewin and colleagues^15^ conceptualized different leadership styles as autocratic, democratic, and laissez-faire. An autocratic leader is similar to how we conceptualize authoritarian leaders, and a democratic leader to an autonomous leader. Although this research provided some insights for our prototype development, our research builds upon this conceptualization by organizing some of these leadership styles under a key underlying factor of control. In this article, we are conceptualizing control as one person *actually influencing* another person to behave in certain ways^16^. An authoritarian leader would be highly controlling, followed by a paternalistic leader, and an autonomous leader would be low on control. The higher the control of the leader, the more immediate social influence they would have over their subordinates. Thus, we expect that authoritarian leaders would influence their subordinates to agree more with fake news.

Past research has also demonstrated the importance of other leadership characteristics, besides the dimension of control. For example, leaders vary in the extent that they are competent or warm^17^. They can also vary in the extent that they are relationship- or task-oriented^18^. And they can also vary in the extent that they would want to transform the culture (i.e., transformational leadership)^19^. To demonstrate the unique contribution of our leadership prototype measure, we included the most relevant leadership characteristics in our study and controlled for them in robustness check analyses (see Supplemental Materials S1).

The present study aimed to explore the relevance of novel leadership prototypes in accuracy and agreeing with fake news in a multinational context. We recruited Prolific workers who had either part-time or full-time jobs and asked them to categorize their immediate superior into one of three different leadership prototypes and characterize them along other relevant leadership dimensions. Subsequently, they reported the accuracy of several fake news articles, then the expectations to agree with it if their superior asked them about it. We aimed to extend both the misinformation and the leadership literature by demonstrating that the social forces of authoritarian leaders are related to people’s belief in fake news more than when people have paternalistic or autonomous leaders. Thus, people may agree with fake news not only because of their inability to cognitively discern real from fake news, but also by conforming to the power of the situation created by authoritarian leaders.

## Methods

### Participants

We collected a sample of 501 respondents from the UK, US, Australia, and Canada in Prolific, an online platform that connects researchers with participants who get paid cash for taking part in the research. The sample was not representative of the UK, US, Australian and Canadian populations with regard to age, sex, and ethnicity. We launched our survey on the 19th of June, 2022, at 10:40 am (GMT + 1). We did not drop any participants as they went through the attention and quality check questions. All participants passed the reCaptcha to check whether there were any bots and we had two English comprehension questions. Although some participants failed these questions, when we reviewed open-ended questions pertaining to the study, their responses were clear and thoughtful. Statistical analyses were conducted on 501 participants (n_US_ = 126; n_Canada_ = 125, n_Australia_ = 125, n_UK_ = 125) who were screened with several questions to gather data only from respondents with part-time (28.5%) or full-time (71.5%) jobs, have the nationality of their respective country (100%), and speak English as their primary language (100%). Participants’ ages ranged from 18 to 73 years of age (*M*_age_ = 36.25 years, *SD*_age_ = 11.88 years). The majority of participants identified as women (60.3%), 38.1% identified as men, and 1.6% identified with another identity. Most of the participants were non-Hispanic White (78.4%), and the remaining 21.6% reported another race (i.e., Black, Asian, Hispanic, Indian, Middle Eastern, Native American, and Pacific Islander). The majority of the participants reported a liberal political ideology (69.5%), 14.2% were conservative, and 16.4% were independent. Almost half of the participants reported their parents or guardians graduated from college with a four-year college degree (43.5%), 53.7% had no college degree, and 2.8% chose not to answer, did not know, or reported the question was not applicable to them. Based on participant responses, 23.8 percent have worked in their current position for 0–1 year, 25.5% for 2–3 years, 17.0% for 4–5 years, 18.4% for 6–10 years, 6.4% for 11–15 years, 5.8% for 16–20 years, and 3.2% for more than 20 years. Finally, 18.8% reported they plan on staying in the same company for 0–1 year, 21.6% for 2–3 years, 19.4% for 4–5 years, 18.4% for 6–10 years, 5.8% for 11–15 years, 5.8% for 16–20 years, and 10.4% for more than 20 years. The study was conducted in accordance with the Declaration of Helsinki and with the approval of The Ohio State University’s ethical committee as well as the informed consent of the participants.

### Measures

#### Autonomous, paternalistic, and authoritarian leader

Inspired by Hazan and Shaver^20^, participants selected the description that best fits the boss or supervisor they interacted with the most (see Table 1 for prototypes). Our objective was to ensure that participants maintained a clearly defined superior in mind while responding to the survey. Thus, after participants selected the description, they were asked to provide a name or nickname of their boss, and we piped that name or nickname to remind participants that they were thinking about a specific boss (and not multiple bosses or bosses in general) as they completed the survey.

**Table 1:** Prototypes of leadership styles and their descriptions.

Please **take your time** and **read carefully** the following descriptions There are many types of bosses. We are going to describe three broad types of bosses. We are not asking you to select who is the best boss, as these types of bosses could be effective in different situations. Instead, we want you to **select the description that best fits the boss or supervisor that you interact most frequently with.** Which of the following best describes your boss? (Please continue scrolling to see all the different types of bosses)
**Autonomous** (*N* = 268, 53.5%): My boss values input from workers, hard work, and creativity. When ideas are discussed, the most important thing is that people bring their best ideas, and they are heard. People who disagree with my boss are encouraged to express their ideas fully and their ideas are respected. The way to be successful is to bring forward good ideas and work hard to implement them. My boss is eager to provide help, and help comes with no strings attached. My boss encourages challenging the system to develop fair and more equitable rules
**Paternalistic** (*N* = 166, 33.1%): My boss values loyalty, hard work, and creativity. When ideas are discussed, people’s voices are heard, but my boss makes the final decision. People who disagree with my boss can still succeed if they follow the rules. There are several ways to be successful (e.g., be loyal, hardworking, or creative), but you have to win over my boss to be successful. My boss is eager to provide help, but receiving help comes with rules that you have to follow. My boss discourages challenging the system, and this can only happen when going through proper channels
**Authoritarian** (*N* = 67, 13.4%): My boss values loyalty over hard work and creativity. When ideas are discussed, the most important thing is that people agree with my boss. People who disagree with my boss have no place in the organization and are cut off from important information. The way to be successful is to agree with my boss. My boss is eager to provide help, but receiving help comes with strings attached. Only my boss or their superiors can change the system, no one else can

#### Accuracy of fake news

Participants were presented with four fake politically-neutral news articles headlines^1^ (e.g., “*The Controversial Files: Fake Cigarettes are Being Sold and Killing People, Here’s How to Spot Counterfeit Packs*”; “*Man Kicked Out Golden Corral After Eating 50LBS of Food; Sues for $2 Million*”). The participants rated the accuracy of each headline using a four-point scale (i.e., “*To the best of your knowledge, how accurate is the claim in the above headline?*”, 1 = *Not at all accurate* to 4 = *Very accurate*).

#### Expectation to agree with fake news

Participants indicated the extent they would be expected to openly agree with their bosses on each news headline (i.e., “*Imagine your boss sent this article to one of your social media accounts, you meet with them, and they ask you about this article. To what extent would you be expected to openly agree with your boss, [piped boss name]?*”) using a five-point scale (1 = *Not at all* to 5 = *Extremely*, *α* = 0.90).

#### Relationship-oriented leadership

Participants indicated the extent that their boss engaged in relationship-oriented behaviors with a four-item measure^18^ (“*My boss tries to make the work fun for others*”, “*My boss helps group members get along*”) using a five-point scale (1 = *Never*, 5 = *Always, α* = 0.87).

#### Task-oriented leadership

Participants indicated the extent that their boss engaged in task-oriented behaviors with a four-item measure (^18^; e.g., *“My boss urges others to concentrate on the work at hand”*, *“My boss sets timelines for when the job needs to be done”*) using a five-point scale (1 = *Never*, 5 = *Always, α* = 0.73).

#### Warmth of leader

Participants indicated the warmth of their boss with a three-item measure (^17^; e.g., *“How friendly is your boss?”*, *“How sincere is your boss?”*) using a five-point scale (*1* = *Not at all, 5* = *Extremely, α* = 0.91).

#### Competence of leader

Participants indicated the competence of their boss with a three-item measure (^17^; e.g., *“How confident is your boss?”*, *“How skillful is your boss?”*) using a five-point scale (*1* = *Not at all, 5* = *Extremely, α* = 0.84).

#### Transformational leadership

Participants indicated the extent that their boss engaged in transformational leadership behaviors with a 10-item measure (^19^; e.g., *“[My boss] challenges me to think about old problems in new ways”*, “*[My boss] says things that make employees proud to be a part of this organization*”) using a five-point scale (1 = *Strongly disagree*, 5 = *Strongly agree, α* = 0.92).

#### Socio-economic status (SES)

SES was assessed with the subjective SES scale from the MacArthur Foundation Research Network on Socioeconomic Status and Health socio-demographic questionnaire (for an example, see Gage-Bouchard et al.^21^; see details in the SM).

#### Political ideology

Political ideology was assessed with a single item measuring the extent that participants identified as liberal to conservative using a seven-point scale (1 = *Extremely liberal*, 7 = *Extremely conservative*).

#### Demographics

Participants reported their gender identity, age, race/ethnicity, and parental education as detailed above.

### Statistical analyses

Statistical analyses and data visualization were performed with R 4.1.1^22^. Within R, the tidyverse^23^, and the ggplot2^24^, packages were used for data transformation and visualization. First, we used ordinary least squares regression models, we examined the effect of the leadership style on fake news agreements. Then, we examined the robustness of the model, by controlling for relevant individual differences such as fake news accuracy ratings, sociodemographic variables, transformational leadership, and competence of the leader.

## Results

### Correlation between accuracy ratings and expectation to agree with fake news

Accuracy ratings and expectation to agree with fake news were weakly positively correlated with each other (*r* = 0.27, *p* < 0.01).

### Main effect of the style of leader on accuracy ratings of fake news

Leadership style led to different accuracy ratings of fake news. Employees with authoritarian leaders rated fake news articles as more accurate than employees with autonomous leaders, (*M*_*Authoritarian*_ = 2.07, *SE*_*Authoritarian*_ = 0.06; *M*_*Autonomous*_ = 1.91, *SE*_*Autonomous*_ = 0.03; b = 0.16, *t* = 2.37, *p* = 0.018, Cohen’s *d* = 0.32). There were no average differences between employees with paternalistic leaders and those with autonomous leaders on accuracy ratings of fake news (*M*_*Paternalistic*_ = 1.98, *SE*_*Paternalistic*_ = 0.04; b = 0.07, *t* = 1.45, *p* = 0.149, Cohen’s *d* = 0.14), neither between employees with authoritarian superiors and those with paternalistic leaders, b = 0.09, *t* = 1.25, *p* = 0.211, Cohen’s *d* = 0.18.

### Main effect of the style of leader on expectation to agree with fake news

**Figure 1:**
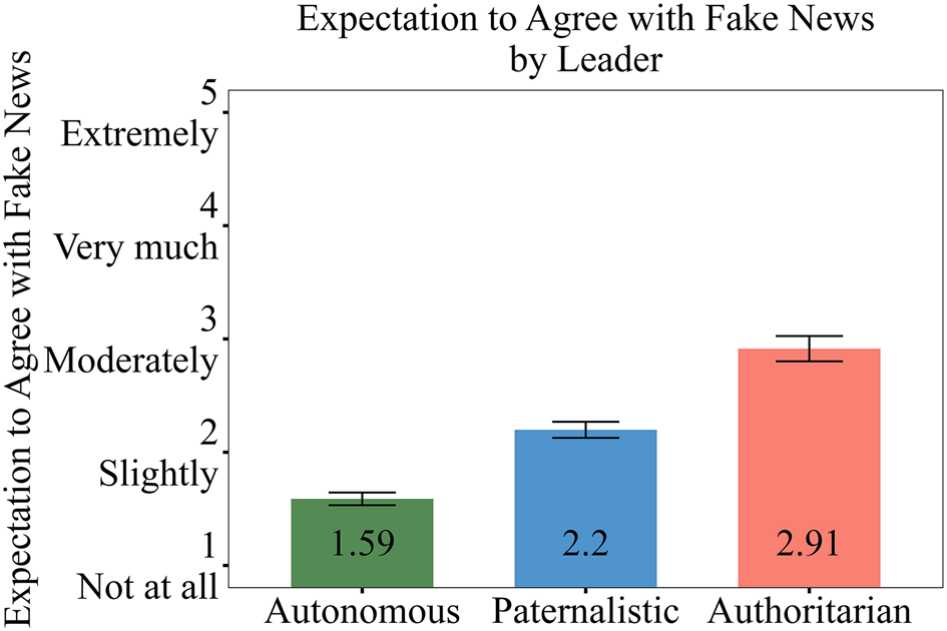
Unstandardized ratings of expectations to agree with misinformation as a function of leadership style, error bars represent standard errors.

Leadership style was related to different expectations to agree with misinformation (see Fig. 1). Employees with authoritarian leaders agreed with fake news more than employees with autonomous leaders, b = 1.33, *t* = 10.62, *p* < 0.001, Cohen’s *d* = 1.30. Employees with paternalistic leaders agreed with fake news more than employees with autonomous leaders, b = 0.61, *t* = 6.75, *p* < 0.001, Cohen’s *d* = 0.60. People with authoritarian superiors agreed with misinformation more than employees with paternalistic leaders, b = 0.72, *t* = 5.41, *p* < 0.001, Cohen’s *d* = 0.70. These effects remained strong and stable, controlling for accuracy ratings, perceived competence, and transformational leadership, and also when controlling for relevant demographics and political ideology (all *p*s < 0.001, for detailed reports of these robustness checks, see the SM).

## Discussion

Our findings provide evidence that social factors are strongly related to agreement with fake news. Specifically, people with authoritarian bosses reported a stronger willingness to agree with misinformation than people with paternalistic or autonomous leaders. People with paternalistic bosses also agreed with fake news more than people with autonomous bosses, these findings are consistent with our argument that the more a boss exercises control over their subordinates, the more those subordinates will agree with fake news promulgated by the boss.

We also found a similar pattern of results of leadership style when we examined accuracy ratings of the fake news, such that employees with authoritarian leaders rated fake news as more accurate compared to employees with autonomous leaders. There was a modest relation between accuracy and agreement with fake news, but the strength of the relation of leadership styles with the accuracy of fake news was weaker (i.e., about a quarter the size) than with agreement of fake news.

In addition, future research should address the extent to which having an authoritarian boss differentially affects agreement with fake news vs believing that fake news is accurate. Our findings are that having an authoritarian boss is more strongly related to agreement with fake news than judgments of accuracy about the fake news. We can imagine, however, that in some situations, having an authoritarian boss might have just as strong or even stronger effects on judgments of accuracy about the fake news than it has on agreement with fake news (i.e., the correlation would be stronger). For example, in situations in which people have come to rely on an authoritarian figure as the sole source of what is true and false, we would expect large effects of that authoritarian figure on both accuracy and agreement with fake news.

This study, however, has several important limitations. First, the samples across different countries were not representative of their populations, so we do not know whether we would find similar trends with a more representative sample. Second, we do not know whether our findings would generalize to other cultures in which authoritarian leaders are more widely present. It is plausible, however, that we would find even stronger effects when studying cultures with more authoritarian regimes. Third, we did not ask participants for their level of education, which may affect the extent that they would believe in misinformation, although we asked them for their parents’ or guardians’ levels of education and other socioeconomic indicators, which when we controlled for these variables did not affect the results (see SM for details of these analyses). Fourth, although the present study was the first to explore the relations between these types of leaders and the tendency of their subordinates to agree with fake news, our data is correlational and thus we cannot address causal claims. Perhaps people who tend to agree with fake news seek out authoritarian or paternalistic bosses. We also cannot rule out that some third variable may cause people to become an authoritarian or paternalistic boss while causing subordinates to be more likely to agree with fake news. Another limitation is that this leadership task assesses perceptions of leaders, and this perception may be biased for multiple reasons (i.e., participants’ upbringing and socialization). For example, people who are raised by authoritarian parents and/or may be socialized with authoritarian superiors may assume that it is important to treat a superior with reverence and respect. Therefore, they may expect to obey any authority, even autonomous leaders, because of their superior position. Despite the correlational nature of our data, we believe it provides and compelling account of accuracy and agreement with fake news that deserves further exploration.

Our research has a considerable number of theoretical and practical implications. Theoretically, this research highlights the potential importance of social influence on agreement with misinformation demonstrating that people may not only fail to reject misinformation when they are less able to distinguish real from fake news, but they may agree with fake news under the pressure of the situation in order to conform to what their (authoritarian) leader wants them to believe. In addition, we developed a novel leadership measure based on common prototypes of leaders and demonstrated that this measure could predict whether people agree with misinformation. If our reasoning is correct that the more leaders exert power over their subordinates, the more those subordinates agree with fake news even when they know it is untrue, then this social influence will likely create pluralistic ignorance^25^ of other’s skepticism of misinformation if people acquiesce to the desires of the leader. People may know that what the leader is saying is wrong but think that they are the only one who thinks this way.

Our research also has many practical implications. To fight misinformation, we not only need to understand how to make people more literate or better able to discern real from fake news, as the cognitive, motivational, and demographic explanations suggest. We also need to teach people how to resist social influence and perhaps puncture pluralistic ignorance. Prior educational interventions nudged people to be more vigilant when they consume news^26–30^. They informed and prepared participants about various ways they can be misinformed (inoculation^31–33^), or targeted media literacy skill development^34–40^. If people understand that news is fake, however, and agree with it nonetheless because of social pressure then none of these interventions will be effective. In these situations, sharpening the cognitive or digital skills are less useful than having wise strategies to fight conformity pressures to reject fake news. From this perspective, we need to implement strategies that could help people find others that help them withstand the social influence of their boss. For instance, we know that a single ally can dramatically reduce conformity in the Asch line judgment study, and we know that when several participants in the Milgram study decided on the shock level together and one of them argued to resist the experimenter, they were much less likely to obey an authority figure. These lessons suggest that if people feel they need to agree with a powerful boss they can resist this social pressure by turning to others and seeking their support. When dealing with a powerful boss who wields that power it may be much more important to curate allies to resist fake news than to recognize it as fake.

The present work opens the door to a wide variety of future research. For example, future studies might examine how norms around publicly accepting fake news emerge under different leadership styles. Future studies may also examine more psychometrically consolidated measures of leadership styles and move beyond the present, relatively simple, prototype leadership measure. Furthermore, our present work is cross-sectional, and we are not using experimental methods to examine causality. Future studies may overcome this shortcoming by systematically manipulating leadership styles and examining how these may impact belief in misinformation.

Also, interventions may be constructed to promote autonomous leadership to decrease belief in misinformation. In addition, future research may differentiate between public acceptance vs. private belief of misinformation. The current study assesses only the expectation to agree with misinformation, but we do not know whether participants are factually believing in these fake news articles (private belief) or if they are complying with the request of their superiors (public acceptance)^41^. Another future direction could be to recruit participants from different societies, not only Western democracies as in the present study. The pattern of results may vary depending on whether participants come from Eastern vs Western societies^42^, individualistic vs interdependent cultures^43^, authoritarian vs democratic regimes^44,45^, or a combination of all these different factors. Another potential direction would be to move beyond leadership in organizations and use this theoretical framework in politics. Future research should also focus on political leaders and their influence on accepting fake news in the context of the recent pandemic^46^.

Importantly, the current findings derive from a hypothetical scenario in which participants imagined their boss approaching them and asking them about the fake news article. What if the situation is not imagined? A tenet of social psychology is the fundamental attribution error, whereby people underestimate the power of the situation and overestimate the power of their own dispositions^47^. For example, most people think they would not obey the authority figure in the Milgram study, and yet we know that most people do^14^. Although the effects we report are quite large we suspect that in real-life situations where a boss has the power to fire subordinates, the social influence of the boss may be even stronger than in this imagined vignette study where the power of the situation may be underestimated. Specifically, we suspect that people with an authoritarian boss will be even less likely to express their disagreement with fake news promulgated by that boss in real life than in our studies.

## Conclusion

We find that when people imagine being asked by their controlling authoritarian boss to agree with headlines that are fake, they express much more agreement with that misinformation than when they are asked to agree with the same news by their autonomous boss. These findings suggest that the power of the social situation may play a much more important role in agreement with misinformation than individual differences or cognitive capacities that have been examined previously. Therefore, a slight change in the focus on what we measure in terms of accuracy vs agreement with misinformation, and the consideration of leadership styles can highlight the role of social psychological factors in both discernment and compliance with misinformation.

### Supplementary Information

Supplementary Information.

## Data Availability

The datasets generated during and/or analyzed during the current study are available from the corresponding author upon reasonable request.
