# Supplementary material for: The relation between authoritarian leadership and belief in fake news
Source: Sci Rep. 2023 Aug 8;13:12860. doi: 10.1038/s41598-023-39807-x (PMC10409744; doi:10.1038/s41598-023-39807-x)
Supplement: Supplementary file 1 — Supplementary Information. [file 41598_2023_39807_MOESM1_ESM.pdf]

The Relation Between Authoritarian Leadership and Belief in Fake News

Juan Ospina, Gábor Orosz, Steven Spencer

Supplemental Materials

Measures

Below, we further describe the SES measure.

Socio-economic status (SES). Subjective SES was assessed with the MacArthur ladder of subjective social status where participants select the rung on which they feel they stand socioeconomically relative to other people in the United States (Adler et al., 2000; e.g., "*Think of this ladder as representing where people stand in the United States. At the TOP of the ladder are the people who are the best off –those who have the most money, the most education, and the most respected jobs. At the BOTTOM are the people who are the worst off–who have the least money, least education, and the least respected jobs or no job. The higher up you are on this ladder, the closer you are to the people at the very top; the lower you are, the closer you are to the people at the very bottom. Where would you place yourself on this ladder? Please indicate the rung where you think you stand at this time in your life, relative to other people in [country]"*) using a 10-point scale (1=*Lowest rung*, 10=*Highest rung*).

Results

Main effect of leadership styles on accuracy ratings

Leadership style was related to different accuracy ratings of fake news (see SM Table 1 and SM Figure 1).

SM Table 1. Coefficients from a linear regression model of the accuracy of fake news as a function of leadership style. The reference group is autonomous leaders. All regression coefficients are unstandardized.

Predictor	b	95% CI	*p*
Paternalistic Leader	.07	[-.025; .167]	.149
Authoritarian Leader	.16	[.028; .293]	.018
R^2	.01		

SM Figure 1. Unstandardized ratings of the accuracy of fake news as a function of leadership style. Error bars represent standard errors.

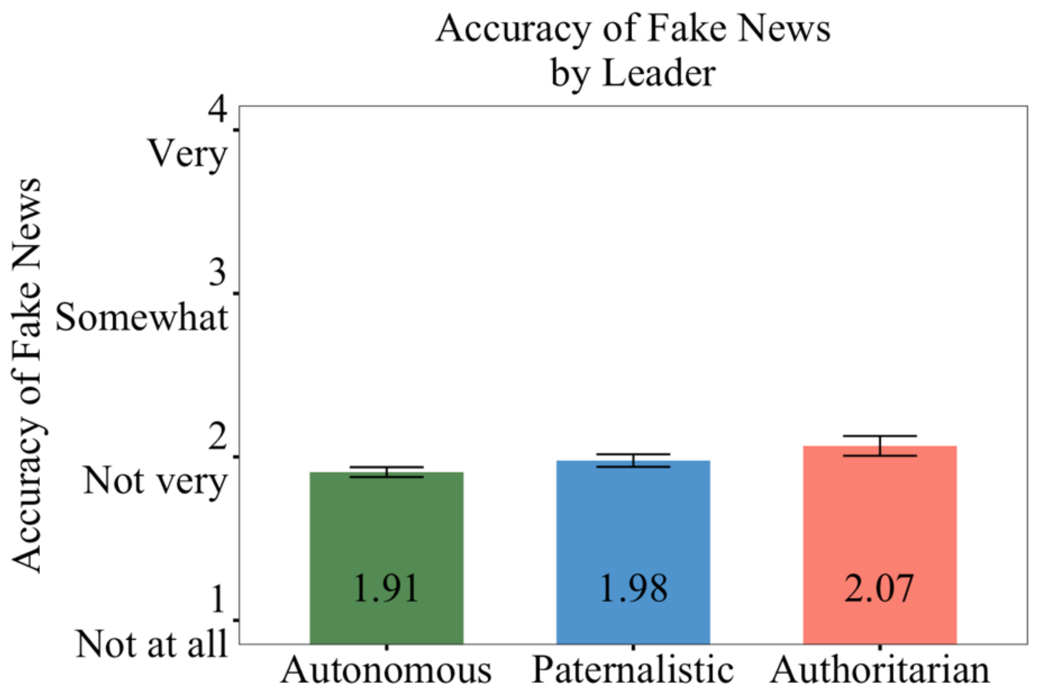

Intraclass correlation coefficient and cultural similarities

Although we recruited participants from four different countries (United States, United Kingdom, Canada, and Australia), we are reporting the results of agreeing with fake news aggregating the data from all these countries because the pattern of results from each country was similar from one country to the other as demonstrated by the low intraclass correlation coefficient (ICC), ICC<0.01. If the ICC was greater than 0.10, this would suggest that each country would be different from each other, and we would have had to control for the random effect of country in multilevel analyses.

Main effect of the style of leader on expectation to agree with fake news

Leadership style was related to different expectations to agree with misinformation (see SM Table 2).

SM Table 2. Coefficients from a linear regression model of the expectations to agree with misinformation as a function of leadership style. The reference group is autonomous leaders. All regression coefficients are unstandardized.

Predictor	b	95% CI	*p*
Paternalistic Leader	.61	[.432; .787]	<.0001
Authoritarian Leader	1.33	[1.081; 1.572]	<.0001
R^2	.21		

Main effect of the style of leader on expectation to agree with fake news controlling for accuracy ratings

To demonstrate the robustness of the effect, leadership style was related to different expectations to agree with misinformation after controlling for accuracy ratings (see SM Table 3 and SM Figure 2). Employees with authoritarian leaders agreed with fake news more than employees with autonomous leaders, b=1.25, t=10.29, p<0.001, Cohen's d=1.22. Employees with paternalistic leaders agreed with fake news more than employees with autonomous leaders, b=0.58, t=6.58, p<0.001, Cohen's d=0.56. Furthermore, people with authoritarian superiors agreed with misinformation more than employees with paternalistic leaders, b=0.68, SE=0.13 t=5.25, p<0.001, Cohen's d=0.66.

SM Table 3. Coefficients from a linear regression model of the expectations to agree with misinformation as a function of leadership style controlling for accuracy ratings of fake news. The reference group is autonomous leaders. All regression coefficients are unstandardized.

Predictor	b	95% CI	*p*
Paternalistic Leader	.58	[.405; .749]	<.0001
Authoritarian Leader	1.25	[1.013; 1.491]	<.0001
Accuracy of Fake News	.46	[.306; .621]	<.0001
R^2	.26		

SM Figure 2. Unstandardized ratings of the expectations to agree with misinformation as a function of leadership style controlling for accuracy ratings of fake news. Error bars represent standard errors.

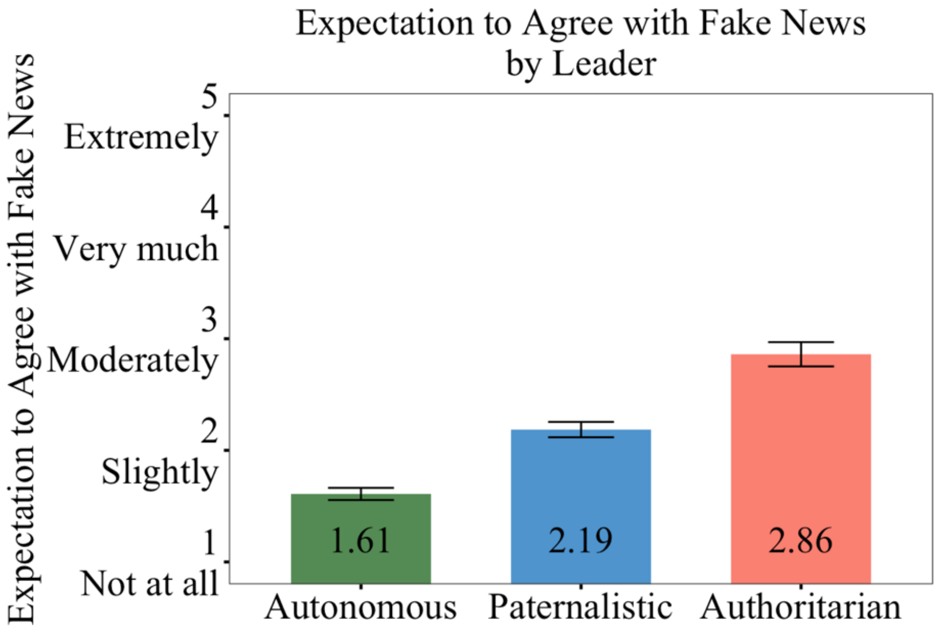

Main effect of the style of leader on the expectation to agree with fake news controlling for accuracy ratings and other leadership characteristics

Moreover, to demonstrate the robustness of the effect, leadership style was related to different expectations to agree with misinformation after controlling for accuracy ratings of fake news, transformational leadership, and competence of the leader (see SM Table 4 and SM Figure 3). Employees with authoritarian leaders agreed with fake news more than employees with autonomous leaders, b=1.063, *t*=7.279, *p*<0.001, Cohen's *d*=1.038, and employees with paternalistic leaders agreed with fake news more than employees with autonomous leaders, b=0.484, *t*=5.021, *p*<0.001, Cohen's *d*=0.473. Finally, people with

authoritarian superiors agreed with misinformation more than employees with paternalistic leaders, b=0.578, t=4.305, p<0.001, Cohen's d=0.565.

SM Table 4. Coefficients from linear regression model of the expectations to agree with misinformation as a function of leadership style controlling for accuracy ratings of fake news, transformational leadership, and competence of the leader. Reference group is autonomous leaders. All regression coefficients are unstandardized.

Predictor	b	95% CI	*p*
Paternalistic Leader	.48	[.295; .674]	<.0001
Authoritarian Leader	1.06	[.776; 1.350]	<.0001
Accuracy of Fake News	.46	[.307; .621]	<.0001
Competence of Leader	.04	[-.077; .160]	.492
Transformational Leadership	-.152	[-.285; -.019]	.025
R^2	.26		

SM Figure 3. Unstandardized ratings of the expectations to agree with misinformation as a function of leadership style controlling for accuracy ratings of fake news, transformational leadership, and competence of the leader. Error bars represent standard errors.

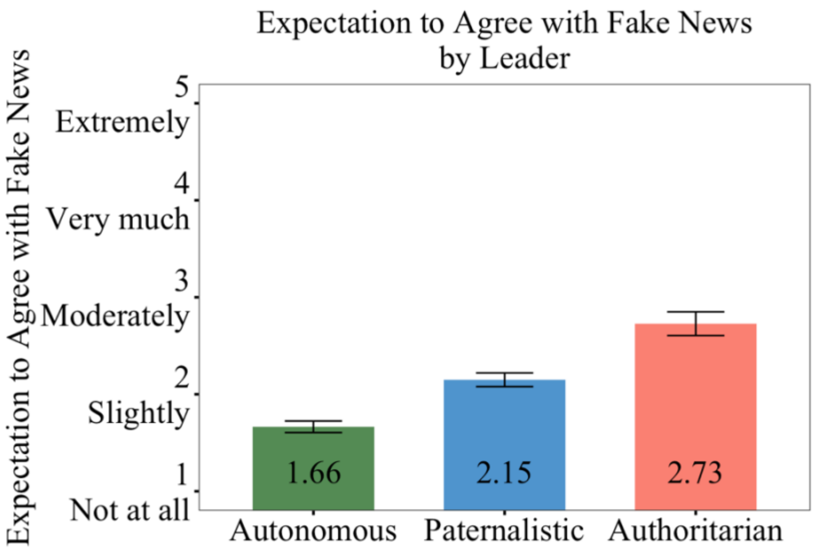

Main effect of the style of leader on expectation to agree with fake news controlling for demographics and political ideology

Moreover, to demonstrate the robustness of the effect, leadership style was related to different expectations to agree with misinformation after controlling for demographics (i.e., age, education of parents, subjective SES, and race), and political ideology (see SM Table 5 and SM Figure 4). Employees with authoritarian leaders agreed with fake news more than employees with autonomous leaders, b=1.301, t=10.343, p<0.001, Cohen's d=1.271, and employees with paternalistic leaders agreed with fake news more than employees with autonomous leaders, b=0.606, t=6.592, p<0.001, Cohen's d=0.592. Finally, people with authoritarian superiors agreed with misinformation more than employees with paternalistic leaders, b=0.694, t=5.249, p<0.001, Cohen's d=0.678.

SM Table 5. Coefficients from a linear regression model of the expectations to agree with misinformation as a function of leadership style controlling for demographics (i.e., age, education of parents, subjective SES, and race), and political ideology. Reference groups are autonomous leaders, no parental education, and White people for leadership styles, parental education, and race, respectively. All regression coefficients are unstandardized.

Predictor	b	95% CI	*p*
Paternalistic Leader	.61	[.426; .787]	<.0001
Authoritarian Leader	1.30	[1.054; 1.548]	<.0001
Age	-.01	[-.016; -.001]	.018
Parental education	.03	[-.141; .200]	.341
Subjective SES	-.04	[-.092; .009]	.108
Political ideology	.09	[.025; .145]	.006
Race	-.01	[-.212; .196]	.940
R^2	.23		

SM Figure 4. Unstandardized ratings of the expectations to agree with misinformation as a function of leadership style and accuracy ratings controlling for age, education of parents, subjective SES, political ideology, and race. Error bars represent standard errors.

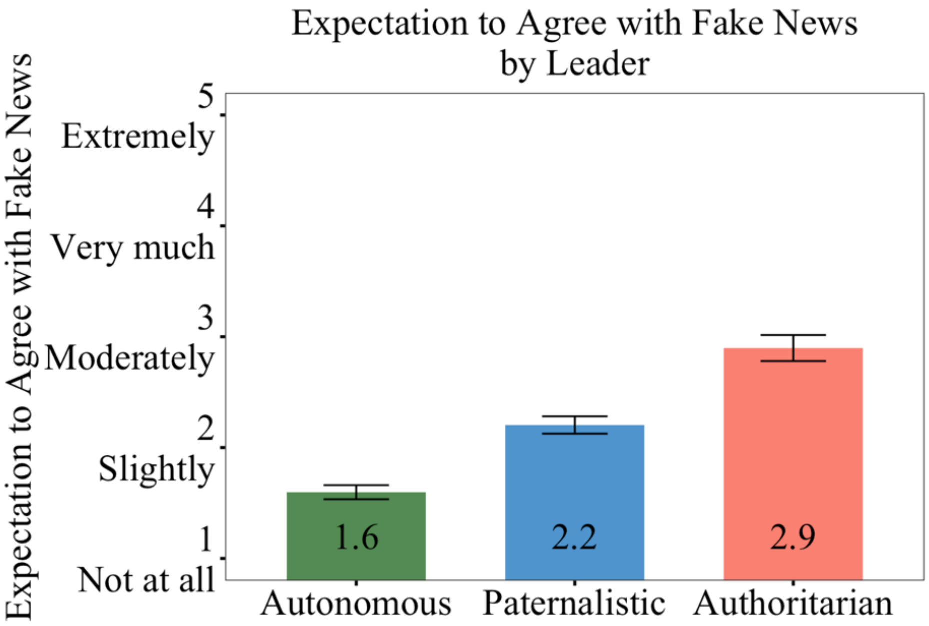

Interactions of leadership styles with other leadership characteristics

We did not control for other leadership characteristics in the models above because we found significant interactions with our leadership styles measure. With a moderation analysis, we examined whether people will be more expected to agree with the authoritarian leader if the supervisor is low on relationship-orientation, high on task-orientation leadership, and low on warmth. Finally, we examined the robustness of the moderating model, by controlling for relevant individual differences such as fake news accuracy ratings, sociodemographic variables, transformational leadership, and competence of the leader.

Interaction with relationship-oriented leadership on expectation to agree with fake news

The strength of the relation of leadership style on agreement with fake news varied with the relationship-orientation of the leader, β=-0.14, t=-2.517, p=0.012 (see SM Table 6 and SM Figure 5). People with authoritarian leaders were more likely to agree with fake news the less relationship-oriented their boss was (simple slope: β=-0.22, t=-2.49, p=0.01). The relation between relationship-oriented leadership style and agreement with fake news, however, was not reliable when people had paternalistic (simple slope: β=-0.08, t=-1.48, p=0.14), or autonomous leaders (simple slope: β=0.07, t=1.04, p=0.30).

SM Table 6. Coefficients from the linear regression model of the expectations to agree with misinformation as a function of relationship-oriented leader interacting with leadership styles.

Leadership styles were effect coded such that autonomous leaders = -1, paternalistic leaders = 0, and authoritarian leaders = 1. All other variables are standardized.

Predictor	*β*	95% CI	*p*
Relationship-oriented Leader	-.08	[-.177; .025]	.140
Leadership Styles	.56	[.422; .700]	<.0001
Relationship-oriented Leader x Leadership Styles	-.14	[-.251; -.031]	.012
R^2	.22		

SM Figure 5. Predicted standardized ratings of expectations to agree with misinformation as a function of leadership style and relationship-orientation leadership (*N*=501), shaded areas represent 95% confidence intervals.

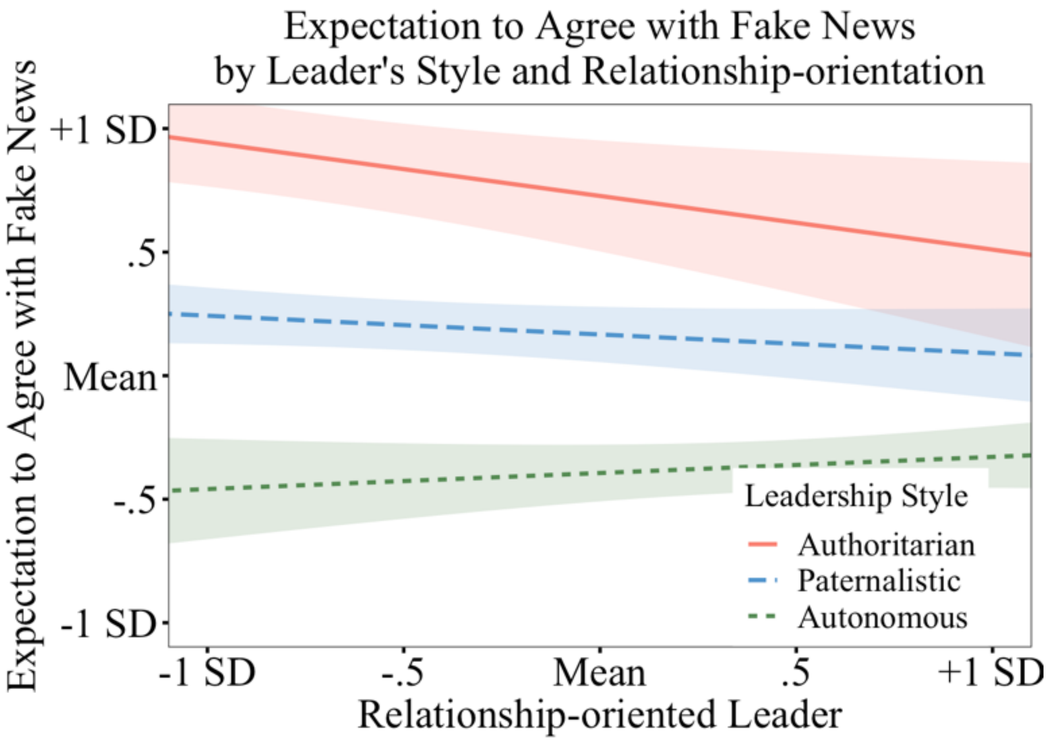

Interaction controlling for accuracy ratings

Moreover, to demonstrate the robustness of the effect, both the main effects of leadership styles and the interaction remained strongly significant after controlling for accuracy ratings of fake news (see SM Table 7).

SM Table 7. Coefficients from a linear regression model of the expectations to agree with misinformation as a function of relationship-oriented leader interacting with leadership styles controlling for accuracy ratings of fake news. Leadership styles were effect coded such that autonomous leaders = -1, paternalistic leaders = 0, and authoritarian leaders = 1. All other variables are standardized.

Predictor	*β*	95% CI	*p*
Relationship-oriented Leader	-.09	[-.183; .013]	.090
Leadership Styles	.52	[.383; .653]	<.0001
Accuracy of Fake News	.23	[.151; .303]	<.0001
Relationship-oriented Leader x Leadership Styles	-.14	[-.249; -.035]	.009
R^2	.27		

Interaction controlling for accuracy and other leadership characteristics

Moreover, to demonstrate the robustness of the effect, both the main effects of leadership styles and the interaction remained strongly significant after controlling for accuracy ratings of fake news, transformational leadership, and competence of the leader (see SM Table 8).

SM Table 8. Coefficients from a linear regression model of the expectations to agree with misinformation as a function of relationship-oriented leader interacting with leadership styles controlling for accuracy ratings of fake news. Leadership styles were effect coded such that autonomous leaders = -1, paternalistic leaders = 0, and authoritarian leaders = 1. All other variables are standardized.

Predictor	*β*	95% CI	*p*
Relationship-oriented Leader	.01	[-.133; .143]	.943
Leadership Styles	.49	[.352; .628]	<.0001
Accuracy of Fake News	.23	[.150; .302]	<.0001
Competence of Leader	.02	[-.084; .120]	.725
Transformational Leadership	-.14	[-.282; -.010]	.068
Relationship-oriented Leader x Leadership Styles	-.12	[-.230; -.012]	.029
R^2	.27		

Interaction controlling for demographics and political ideology

Moreover, to demonstrate the robustness of the effect, both the main effects of leadership styles and the interaction remained strongly significant after controlling for demographics (i.e., age, education of parents, subjective SES, and race/ethnicity) and political ideology (see SM Table 9).

SM Table 9. Coefficients from linear regression model of the expectations to agree with misinformation as a function of relationship-oriented leader interacting with leadership styles controlling for demographics (i.e., age, education of parents, subjective SES, and race), and political ideology. Reference groups are no parental education, and White people for parental education, and race, respectively. Leadership styles were effect coded such that autonomous leaders = -1, paternalistic leaders = 0, and authoritarian leaders = 1. All other variables are standardized.

Predictor	β	95% CI	p
Relationship-oriented Leader	-.10	[-.202; .002]	.056
Leadership Styles	.53	[.392; .672]	<.0001
Age	-.10	[-.187; -.020]	.015
Parental education	.04	[-.130; .202]	.670
Subjective SES	-.07	[-.150; .013]	.102
Political ideology	.12	[.040; .198]	.003
Race	.02	[-.183; .218]	.862
Relationship-oriented Leader x Leadership Styles	-.15	[-.260; -.039]	.008
R^2	.25		

Interaction with warmth of leader on expectation to agree with fake news

The strength of the relation of leadership style on agreement with fake news varied with the warmth of the leader, β=-0.18, t=-3.274, p=0.001 (see SM Table 10 and SM Figure 6). People with authoritarian leaders were more likely to agree with fake news the less warm their boss was (simple slope: β=-0.39, t=-4.73, p<0.01), followed by paternalistic leaders (simple slope: β=-0.21, t=-4.34, p<0.01). The relation between the warmth of leader and agreement with fake news, however, was not reliable when people had autonomous leaders (simple slope: β=-0.03, t=-0.47, p=0.64).

SM Table 10. Coefficients from linear regression model of the expectations to agree with misinformation as a function of leader's warmth interacting with leadership styles. Leadership styles were effect coded such that autonomous leaders = -1, paternalistic leaders = 0, and authoritarian leaders = 1. All other variables are standardized.

Predictor	β	95% CI	p
Leader's Warmth	-.21	[-.307; -.115]	<.0001
Leadership Styles	.45	[.321; .586]	<.0001
Leader's Warmth x Leadership Styles	-.18	[-.291; -.073]	.001
R^2	.24		

SM Figure 6. Predicted standardized ratings of expectations to agree with misinformation as a function of leadership style and warmth of the leadership, controlling for accuracy ratings of the fake news (N=501), shaded areas represent 95% confidence intervals.

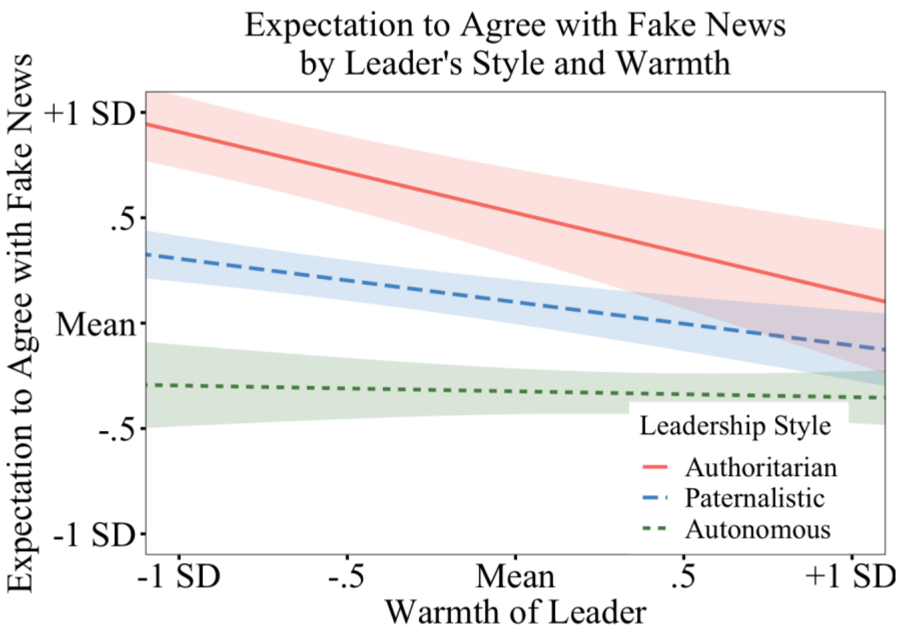

Interaction controlling for accuracy ratings

Moreover, to demonstrate the robustness of the effect, both the main effects of leadership styles and the interaction remained strongly significant after controlling for accuracy ratings of fake news (see SM Table 11).

SM Table 11. Coefficients from linear regression model of the expectations to agree with misinformation as a function of leader's warmth interacting with leadership styles controlling for accuracy ratings of fake news. Leadership styles were effect coded such that autonomous leaders = -1, paternalistic leaders = 0, and authoritarian leaders = 1. All other variables are standardized.

Predictor	*β*	95% CI	*p*
Leader's Warmth	-.21	[-.298; -.113]	<.0001
Leadership Styles	.42	[.295; .552]	<.0001
Accuracy of Fake News	.22	[.146; .296]	<.0001
Leader's Warmth x Leadership Styles	-.18	[-.284; -.072]	.001
R^2	.29		

Interaction controlling for accuracy ratings and other leadership characteristics

Moreover, to demonstrate the robustness of the effect, both the main effects of leadership styles and the interaction remained strongly significant after controlling for fake news accuracy ratings, perceived competence and transformational leadership (see SM Table 12).

SM Table 12. Coefficients from linear regression model of the expectations to agree with misinformation as a function of leader's warmth interacting with leadership styles controlling for accuracy ratings of fake news. Leadership styles were effect coded such that autonomous leaders = -1, paternalistic leaders = 0, and authoritarian leaders = 1. All other variables are standardized.

Predictor	*β*	95% CI	*p*
Leader's Warmth	-.23	[-.351; -.108]	<.0001
Leadership Styles	.42	[.288; .559]	<.0001
Accuracy of Fake News	.22	[.144; .294]	<.0001
Competence of Leader	.06	[-.044; .163]	.257
Transformational Leadership	-.02	[-.146; -.113]	.805
Leader's Warmth x Leadership Styles	-.17	[-.280; -.066]	. 002
R^2	.29		

Interaction controlling for demographics and political ideology

Moreover, to demonstrate the robustness of the effect, both the main effects of leadership styles and the interaction remained strongly significant after controlling for demographics and political ideology (see SM Table 13).

SM Table 13. Coefficients from the linear regression model of the expectations to agree with misinformation as a function of leader's warmth interacting with leadership styles controlling for demographics (i.e., age, education of parents, subjective SES, and race), and political ideology. Reference groups are no parental education, and White people for parental education, and race, respectively. Leadership styles were effect coded such that autonomous leaders = -1, paternalistic leaders = 0, and authoritarian leaders = 1. All other variables are standardized.

Predictor	*β*	95% CI	*p*
Leader's Warmth	-.23	[-.330; -.137]	<.0001
Leadership Styles	.427	[.293; .560]	<.0001
Age	-.11	[-.187; -.023]	.012
Parental education	.05	[-.113; .212]	.551
Subjective SES	-.06	[-.143; .017]	.121
Political ideology	.13	[.054; .210]	.001
Race	-.02	[-.215; .175]	.837
Leader's Warmth x Leadership Styles	-.19	[-.298; -.080]	. 001
R^2	.28		

Interaction with task-oriented leadership on expectation to agree with fake news

The strength of the relation of leadership style on agreement with fake news varied with task-orientation leadership, β=0.12, t=2.362, p=0.019 (see SM Table 14 and SM Figure 7). People with authoritarian leaders were more likely to agree with fake news the more task-oriented their boss was (simple slope: β=0.25, t=3.27, p<0.01), followed by paternalistic leader (simple slope: β=0.13, t=3.06, p<0.01). The relation between task-oriented leadership and agreement with fake news, however, was not reliable when people had autonomous leaders (simple slope: β=0.01, t=0.16, p=0.88).

SM Table 14. Coefficients from linear regression model of the expectations to agree with misinformation as a function of task-oriented leader interacting with leadership styles. Leadership styles were effect coded such that autonomous leaders = -1, paternalistic leaders = 0, and authoritarian leaders = 1. All other variables are standardized.

Predictor	β	95% CI	p
Task-oriented Leader	.13	[.046; .211]	.002
Leadership Styles	.64	[.529; .747]	<.0001
Task-oriented Leader x Leadership Styles	.12	[.020; .220]	.019
R^2	.22		

SM Figure 7. Predicted standardized ratings of expectations to agree with misinformation as a function of leadership style and task-oriented leadership, controlling for accuracy ratings of the fake news (N=501), shaded areas represent 95% confidence intervals.

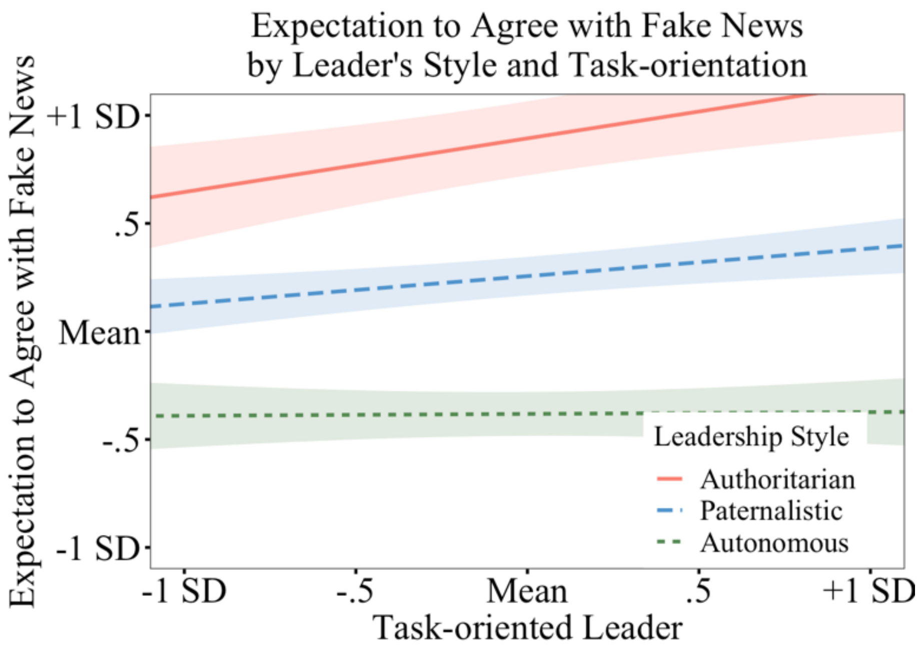

Interaction controlling for accuracy ratings

Moreover, to demonstrate the robustness of the effect, both the main effects of leadership styles and the interaction remained strongly significant after controlling for accuracy ratings of fake news (see SM Table 15).

SM Table 15. Coefficients from linear regression model of the expectations to agree with misinformation as a function of task-oriented leader interacting with leadership styles controlling for accuracy ratings of fake news. Leadership styles were effect coded such that autonomous leaders = -1, paternalistic leaders = 0, and authoritarian leaders = 1. All other variables are standardized.

Predictor	β	95% CI	p
Task-oriented Leader	.12	[.043; .202]	.003
Leadership Styles	.60	[.497; .709]	<.0001
Accuracy of Fake News	.22	[.146; .298]	<.0001
Task-oriented Leader x Leadership Styles	.12	[.021; .214]	.017
R^2	.27		

Interaction controlling for accuracy ratings and other leadership characteristics

Moreover, to demonstrate the robustness of the effect, both the main effects of leadership styles and the interaction remained strongly significant after controlling for fake news accuracy ratings, perceived competence and transformational leadership (see SM Table 16).

SM Table 16. Coefficients from linear regression model of the expectations to agree with misinformation as a function of task-oriented leader interacting with leadership styles controlling for accuracy ratings of fake news. Leadership styles were effect coded such that autonomous leaders = -1, paternalistic leaders = 0, and authoritarian leaders = 1. All other variables are standardized.

Predictor	β	95% CI	p
Task-oriented Leader	.16	[.078; .247]	<.0001
Leadership Styles	.47	[.338; .605]	<.0001
Accuracy of Fake News	.22	[.148; .299]	<.0001
Competence of Leader	.003	[-.099; .104]	.961
Transformational Leadership	-.16	[-.276; -.044]	.007
Task-oriented Leader x Leadership Styles	.11	[.017; .209]	.021
R^2	.29		

Interaction controlling for demographics and political ideology

Moreover, to demonstrate the robustness of the effect, both the main effects of leadership styles and the interaction remained strongly significant after controlling for demographics and political ideology (see SM Table 17).

SM Table 17. Coefficients from linear regression model of the expectations to agree with misinformation as a function of task-oriented leader interacting with leadership styles controlling for demographics (i.e., age, education of parents, subjective SES, and race), and political ideology. Reference groups are no parental education, and White people for parental education, and race, respectively. Leadership styles were effect coded such that autonomous leaders = -1, paternalistic leaders = 0, and authoritarian leaders = 1. All other variables are standardized.

Predictor	*β*	95% CI	*p*
Task-oriented Leader	.11	[.026; .193]	.010
Leadership Styles	.63	[.519; .741]	<.0001
Age	-.10	[-.187; -.021]	.015
Parental education	.01	[-.153; .179]	.875
Subjective SES	-.06	[-.139; .025]	.174
Political ideology	.11	[.033; .191]	.005
Race	-.01	[-.211; .185]	.896
Task-oriented Leader x Leadership Styles	.12	[.019; .219]	.020
R^2	.25		
